# Supplementary material for: Functional Characteristics of Serum Anti-SARS-CoV-2 Antibodies against Delta and Omicron Variants after Vaccination with Sputnik V
Source: Viruses. 2023 Jun 10;15(6):1349. doi: 10.3390/v15061349 (PMC10301317; doi:10.3390/v15061349)
Supplement: Supplementary file 1 [file viruses-15-01349-s001.zip › viruses-2418566-supplementary.pdf]

A.

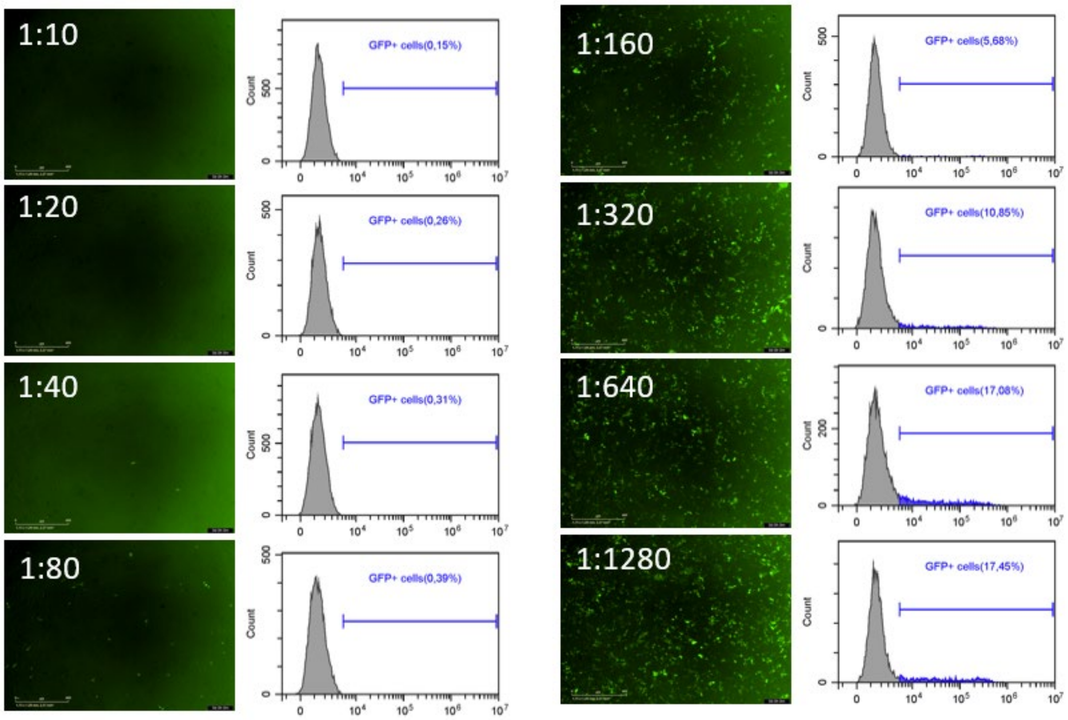

B.

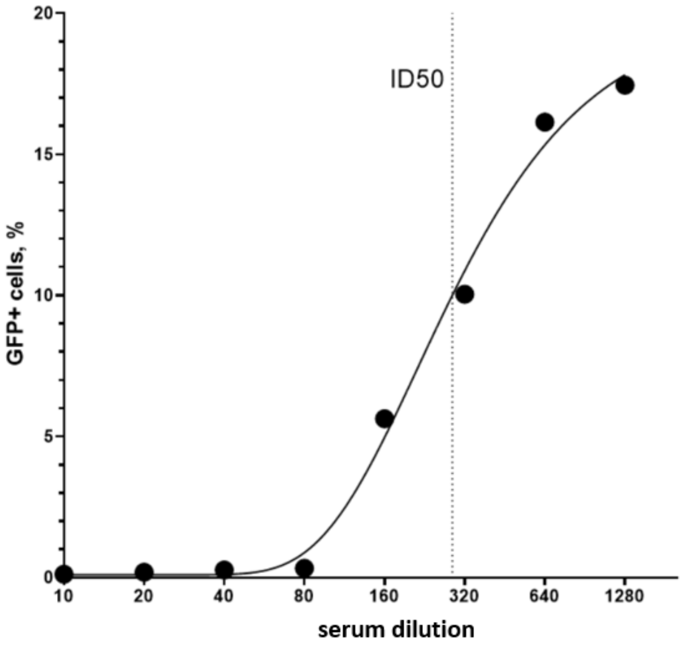

Figure S1. Representative plots of pseudotyped lentiviral neutralization assay. (**A**) Fluorescence microscopy and flow cytometry data of the representative experiment of HEK293T-hACE2-TMPRSS2 cells transduced with lentiviral particles pseudotyped with the S-protein of the SARS-CoV-2 variant D614G in the presence of patient sera. Serum sample dilutions are shown in white in the upper left corner of each figure section. (**B**) Graphical representation of ID50 calculation using a non-linear regression model. The Y-axis shows the percent of GFP-positive cells. The X-axis shows the corresponding dilution of patient sera.

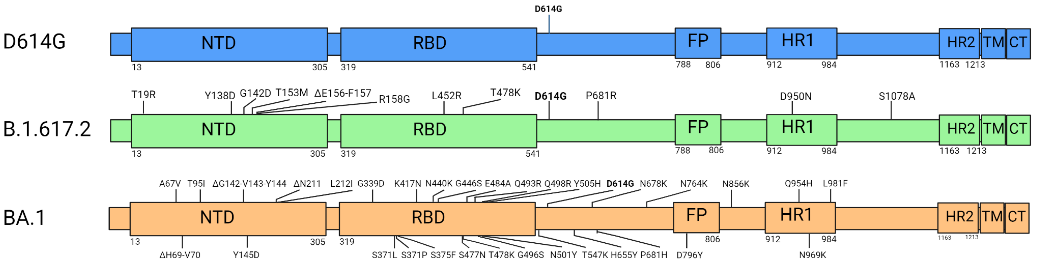

Figure S2. Schematic representation of amino acid substitutions in S-proteins of SARS-CoV-2 variants D614G, B.1.617.2, and BA.1. D614G refers to the Wuhan-Hu-1 SARS-CoV-2 G614-variant. This variant is characterized by the D614G mutation, which is associated with increased infectivity of COVID-19 [13].

Appendix A: Nucleotide sequences of 19-aminoacids-truncated S-proteins used in the study.

B1. S-protein D614G.

ATGTTCGTGTTTCTGGTGCTGCTGCCTCTGGTGTCCAGCCAGTGTGTGAACCTGACCACCAGAACACAGCTGCCTCCAGCCTACACCA
ACAGCTTTACCAGGGGCGTGTACTACCCCGACAAGGTGTTCAGATCCAGCGTGCTGCACTCTACCCAGGACCTGTTCCTGCCTTTCTTC
AGCAACGTGACCTGGTTCCACGCCATTCACGTGTCCGGCACCAATGGCACCAAGAGATTCGACAACCCCGTGCTGCCCTTCAACGAC
GGGGTGTACTTTGCCAGCACCGAGAAGTCCAACATCATCCGCGGCTGGATCTTCGGCACCACACTGGATAGCAAGACCCAGAGCCTG
CTGATCGTGAACAACGCCACCAACGTGGTCATCAAAGTGTGCGAGTTCCAGTTCTGCAACTACCCCTTCCTGGGCGTCTACTATCACA
AGAACAACAAGAGCTGGACCGAGAGCGAGTTCCGGGTGTACAGCAGCGCCAACAACTGCACCTTCGAGTACGTGTCCCAGCCTTTCC
TGATGGACCTGGAAGGCAAGCAGGGCAACTTCAAGAACCTGCGCGAGTTCGTGTTCAAGAACATCGACGGCTACTTCAAGATCTACA
GCAAGCACACCCCTATCAACCTCGTGCGGGATCTGCCTCAGGGCTTCTCTGCTCTGGAACCCCTGGTGGATCTGCCCATCGGCATCAA
CATCACCCGGTTTCAGACACTGCTGGCCCTGCACAGAAGCTACCTGACACCTGGCGATAGCAGCAGCGGATGGACAGCTGGTGCCGC
CGCTTACTATGTGGGCTACCTGCAGCCTAGAACCTTCCTGCTGAAGTACAACGAGAACGGCACCATCACCGACGCCGTGGATTGTGCT
CTGGATCCTCTGAGCGAGACAAAGTGCACCCTGAAGTCCTTCACCGTGGAAAAGGGCATCTACCAGACCAGCAACTTCCGGGTGCAG
CCCACCGAATCCATCGTGCGGTTCCCCAATATCACCAATCTGTGCCCCTTCGGCGAGGTGTTCAATGCCACCAGATTCGCCTCTGTGTA
CGCCTGGAACCGGAAGCGGATCAGCAATTGCGTGGCCGACTACTCCGTGCTGTACAATAGCGCCAGCTTCAGCACCTTCAAGTGCTA
CGGCGTGTCCCCTACCAAGCTGAACGACCTGTGCTTCACAAACGTGTACGCCGACAGCTTCGTGATCCGGGGAGATGAAGTGCGGCA
GATTGCCCCTGGACAGACAGGCAAGATCGCCGACTACAACTACAAGCTGCCCGACGACTTCACCGGCTGCGTGATCGCTTGGAACAG
CAACAACCTGGACAGCAAAGTCGGCGGCAACTACAATTACCTGTACCGGCTGTTCCGGAAGTCCAATCTGAAGCCCTTCGAGCGGGA
CATCTCCACCGAGATCTATCAGGCCGGCAGCACCCCTTGTAACGGCGTGGAAGGCTTCAACTGCTACTTCCCACTGCAGTCCTACGGC
TTTCAGCCCACAAATGGCGTCGGCTACCAGCCTTACAGAGTGGTGGTGCTGAGCTTCGAGCTGCTGCATGCTCCTGCCACAGTGTGCG
GCCCTAAGAAAAGCACCAATCTCGTGAAGAACAAATGCGTGAACTTCAACTTCAACGGCCTGACCGGCACCGGCGTGCTGACAGAG
AGCAACAAGAAGTTCCTGCCATTCCAGCAGTTCGGCCGGGATATCGCCGATACCACAGATGCCGTCAGAGATCCCCAGACACTGGA
AATCCTGGACATCACCCCTTGCAGCTTCGGCGGAGTGTCTGTGATCACCCCTGGCACCAACACCAGCAATCAGGTGGCAGTGCTGTAC
CAGGGCGTGAACTGTACAGAGGTGCCAGTGGCCATCCATGCCGATCAGCTGACCCCTACTTGGAGAGTGTACTCCACCGGCAGCAAT
GTGTTTCAGACCAGAGCCGGCTGTCTGATCGGAGCCGAGCACGTGAACAATAGCTACGAGTGCGACATCCCCATCGGCGCTGGCATC
TGCGCCTCTTACCAGACACAGACAAACAGCCCCAGACGGGCCAGATCTGTGGCCAGCCAGAGCATCATTGCCTACACAATGTCTCTG
GGCGCCGAGAACTCTGTGGCCTACTCCAACAACTCTATCGCTATCCCCACCAACTTCACCATCAGCGTGACCACAGAGATCCTGCCTG
TGTCCATGACCAAGACCAGCGTGGACTGCACCATGTACATCTGCGGCGATTCCACCGAGTGCTCCAACCTGCTGCTGCAGTACGGCA
GCTTCTGCACCCAGCTGAATAGAGCCCTGACAGGGATCGCCGTGGAACAGGACAAGAACACCCAAGAGGTGTTCGCCCAAGTGAAG
CAGATCTACAAGACCCCTCCTATCAAGGACTTCGGCGGCTTCAATTTCAGCCAGATTCTGCCCGATCCTAGCAAGCCCAGCAAGCGG
AGCTTCATCGAGGACCTGCTGTTCAACAAAGTGACACTGGCCGACGCCGGCTTCATCAAGCAGTATGGCGATTGCCTGGGCGACATT
GCCGCCAGGGATCTGATTTGCGCCCAGAAGTTTAACGGACTGACAGTGCTGCCTCCTCTGCTGACCGATGAGATGATCGCCCAGTATA
CAAGCGCCCTGCTGGCCGGCACAATCACAAGCGGCTGGACATTTGGAGCTGGCGCCGCTCTGCAGATCCCCTTTGCTATGCAGATGG
CCTACAGATTCAACGGCATCGGAGTGACCCAGAATGTGCTGTACGAGAACCAGAAGCTGATCGCCAACCAGTTCAACAGCGCCATC
GGCAAGATCCAGGACAGCCTGAGCAGTACAGCCAGCGCTCTGGGAAAGCTGCAGGACGTGGTCAACCAGAATGCCCAGGCACTGA
ACACCCTGGTCAAGCAGCTGAGCAGCAATTTCGGCGCCATCAGCTCTGTGCTGAACGATATCCTGAGCAGACTGGACAAGGTGGAAG
CCGAGGTGCAGATCGACAGACTGATCACCGGAAGGCTGCAGTCCCTGCAGACCTACGTTACCCAGCAGCTGATCAGAGCCGCCGAG
ATTAGAGCCTCTGCCAATCTGGCCGCCACAAAGATGAGCGAGTGTGTGCTGGGCCAGAGCAAGAGAGTGGACTTCTGCGGAAAGGG
CTACCACCTGATGAGCTTCCCTCAGTCTGCACCACACGGCGTGGTGTTTCTGCACGTGACATACGTGCCCGCTCAAGAGAAGAACTTC
ACAACAAGCCCCGCCATCTGCCACGACGGCAAAGCCCACTTTCCTAGAGAAGGCGTGTTCGTGTCCAACGGCACCCATTGGTTCGTG

ACCCAGCGGAACTTCTACGAGCCCCAGATCATCACCACCGACAACACCTTCGTGTCTGGCAACTGCGACGTCGTGATCGGAATTGTG
AACAATACCGTGTACGACCCTCTGCAGCCCGAGCTGGACAGCTTCAAAGAGGAACTGGATAAGTACTTTAAGAACCACACTAGCCCC
GACGTGGACCTGGGCGATATCAGCGGAATCAATGCCAGCGTCGTGAACATCCAGAAAGAGATCGACCGGCTGAACGAGGTGGCCAA
GAATCTGAACGAGAGCCTGATCGACCTGCAAGAACTGGGGAAGTACGAGCAGTATATTAAGTGGCCTTGGTACATCTGGCTGGGCTT
TATCGCCGGACTGATTGCCATCGTGATGGTCACAATCATGCTGTGCTGCATGACCAGCTGCTGTAGCTGCCTGAAGGGCTGTTGTAGC
TGTGGCTCCTGCTGCTGATAG

S-proteinB.1.617.2

ATGTTCGTGTTTCTGGTGCTGCTGCCTCTGGTGTCCAGCCAGTGTGTGAACCTGCGGACCAGAACACAGCTGCCTCCAGCCTACACCA
ACAGCTTTACCAGGGGCGTGTACTACCCCGACAAGGTGTTCAGATCCAGCGTGCTGCACTCTACCCAGGACCTGTTCCTGCCTTTCTTC
AGCAACGTGACCTGGTTCCACGCCATTCACGTGTCCGGCACCAATGGCACCAAGAGATTCGACAACCCCGTGCTGCCCTTCAACGAC
GGGGTGTACTTTGCCAGCACCGAGAAGTCCAACATCATCCGCGGCTGGATCTTCGGCACCACACTGGATAGCAAGACCCAGAGCCTG
CTGATCGTGAACAACGCCACCAACGTGGTCATCAAAGTGTGCGAGTTCCAGTTCTGCAACGACCCTTTCCTGGACGTGTACTATCACA
AGAACAACAAGAGCTGGATGGAAAGCGGCGTGTACAGCAGCGCCAACAACTGCACCTTCGAGTACGTGTCCCAGCCTTTCCTGATG
GACCTGGAAGGCAAGCAGGGCAACTTCAAGAACCTGCGCGAGTTCGTGTTCAAGAACATCGACGGCTACTTCAAGATCTACAGCAA
GCACACCCCTATCAACCTCGTGCGGGATCTGCCTCAGGGCTTCTCTGCTCTGGAACCCCTGGTGGATCTGCCCATCGGCATCAACATC
ACCCGGTTTCAGACACTGCTGGCCCTGCACAGAAGCTACCTGACACCTGGCGATAGCAGCAGCGGATGGACAGCTGGTGCCGCCGCT
TACTATGTGGGCTACCTGCAGCCTAGAACCTTCCTGCTGAAGTACAACGAGAACGGCACCATCACCGACGCCGTGGATTGTGCTCTG
GATCCTCTGAGCGAGACAAAGTGCACCCTGAAGTCCTTCACCGTGGAAAAGGGCATCTACCAGACCAGCAACTTCCGGGTGCAGCCC
ACCGAATCCATCGTGCGGTTCCCCAATATCACCAATCTGTGCCCCTTCGGCGAGGTGTTCAATGCCACCAGATTCGCCTCTGTGTACG
CCTGGAACCGGAAGCGGATCAGCAATTGCGTGGCCGACTACTCCGTGCTGTACAATAGCGCCAGCTTCAGCACCTTCAAGTGCTACG
GCGTGTCCCCTACCAAGCTGAACGACCTGTGCTTCACAAACGTGTACGCCGACAGCTTCGTGATCCGGGGAGATGAAGTGCGGCAGA
TTGCCCCTGGACAGACAGGCAAGATCGCCGACTACAACTACAAGCTGCCCGACGACTTCACCGGCTGCGTGATCGCTTGGAACAGCA
ACAACCTGGACAGCAAAGTCGGCGGCAACTACAATTACAGATACCGGCTGTTCCGGAAGTCCAATCTGAAGCCCTTCGAGCGGGAC
ATCTCCACCGAGATCTATCAGGCCGGCAGCAAGCCTTGCAACGGCGTGGAAGGCTTCAACTGCTACTTCCCACTGCAGTCCTACGGCT
TTCAGCCCACAAATGGCGTCGGCTACCAGCCTTACAGAGTGGTGGTGCTGAGCTTCGAGCTGCTGCATGCTCCTGCCACAGTGTGCGG
CCCTAAGAAAAGCACCAATCTCGTGAAGAACAAATGCGTGAACTTCAACTTCAACGGCCTGACCGGCACCGGCGTGCTGACAGAGA
GCAACAAGAAGTTCCTGCCATTCCAGCAGTTCGGCCGGGATATCGCCGATACCACAGATGCCGTCAGAGATCCCCAGACACTGGAA
ATCCTGGACATCACCCCTTGCAGCTTCGGCGGAGTGTCTGTGATCACCCCTGGCACCAACACCAGCAATCAGGTGGCAGTGCTGTACC
AGGGCGTGAACTGTACAGAGGTGCCAGTGGCCATCCATGCCGATCAGCTGACCCCTACTTGGAGAGTGTACTCCACCGGCTCCAACG
TGTTCCAGACAAGAGCCGGCTGTCTGATCGGAGCCGAGCACGTGAACAATAGCTACGAGTGCGACATCCCCATCGGCGCTGGCATCT
GCGCCTCTTACCAGACACAGACCAACAGCAGGCGGAGAGCCAGATCTGTGGCCAGCCAGAGCATCATTGCCTACACAATGTCTCTGG
GCGCCGAGAACTCTGTGGCCTACTCCAACAACTCTATCGCTATCCCCACCAACTTCACCATCAGCGTGACCACAGAGATCCTGCCTGT
GTCCATGACCAAGACCAGCGTGGACTGCACCATGTACATCTGCGGCGATTCCACCGAGTGCTCCAACCTGCTGCTGCAGTACGGCAG
CTTCTGCACCCAGCTGAATAGAGCCCTGACAGGGATCGCCGTGGAACAGGACAAGAACACCCAAGAGGTGTTCGCCCAAGTGAAGC
AGATCTACAAGACCCCTCCTATCAAGGACTTCGGCGGCTTCAATTTCAGCCAGATTCTGCCCGATCCTAGCAAGCCCAGCAAGCGGA
GCTTCATCGAGGACCTGCTGTTCAACAAAGTGACACTGGCCGACGCCGGCTTCATCAAGCAGTATGGCGATTGCCTGGGCGACATTG
CCGCCAGGGATCTGATTTGCGCCCAGAAGTTTAACGGACTGACAGTGCTGCCTCCTCTGCTGACCGATGAGATGATCGCCCAGTATAC
AAGCGCCCTGCTGGCCGGCACAATCACAAGCGGCTGGACATTTGGAGCTGGCGCCGCTCTGCAGATCCCCTTTGCTATGCAGATGGC
CTACAGATTCAACGGCATCGGAGTGACCCAGAATGTGCTGTACGAGAACCAGAAGCTGATCGCCAACCAGTTCAACAGCGCCATCG
GCAAGATCCAGGACAGCCTGAGCAGTACAGCCAGCGCTCTGGGCAAGCTGCAGAACGTCGTGAACCAGAATGCCCAGGCACTGAAC
ACCCTGGTCAAGCAGCTGAGCAGCAATTTCGGCGCCATCAGCTCTGTGCTGAACGATATCCTGAGCAGACTGGACAAGGTGGAAGCC
GAGGTGCAGATCGACAGACTGATCACCGGAAGGCTGCAGTCCCTGCAGACCTACGTTACCCAGCAGCTGATCAGAGCCGCCGAGAT
TAGAGCCTCTGCCAATCTGGCCGCCACAAAGATGAGCGAGTGTGTGCTGGGCCAGAGCAAGAGAGTGGACTTCTGCGGAAAGGGCT
ACCACCTGATGAGCTTCCCTCAGTCTGCACCACACGGCGTGGTGTTTCTGCACGTGACATACGTGCCCGCTCAAGAGAAGAATTTCAC
CACCGCTCCAGCCATCTGCCACGACGGCAAAGCCCACTTTCCTAGAGAAGGCGTGTTCGTGTCCAACGGCACCCATTGGTTCGTGACC
CAGCGGAACTTCTACGAGCCCCAGATCATCACCACCGACAACACCTTCGTGTCTGGCAACTGTGACGTCGTGATCGGGATTGTGAAC
AATACCGTGTACGACCCTCTGCAGCCCGAGCTGGACAGCTTCAAAGAGGAACTGGATAAGTACTTTAAGAACCACACAAGCCCCGA
CGTGGACCTGGGCGATATCAGCGGAATCAATGCCAGCGTGGTCAACATCCAGAAAGAGATCGACCGGCTGAACGAGGTGGCCAAGA
ATCTGAACGAGAGCCTGATCGACCTGCAAGAACTGGGGAAGTACGAGCAGTATATTAAGTGGCCTTGGTACATCTGGCTGGGCTTTA
TCGCCGGACTGATTGCCATCGTGATGGTCACAATCATGCTGTGCTGCATGACCAGCTGCTGTAGCTGCCTGAAGGGCTGTTGTAGCTG
TGGCTCCTGCTGCTGATAG

S-protein BA.1

ATGTTCGTGTTTCTGGTGCTGCTGCCTCTGGTGTCCAGCCAGTGTGTGAACCTGACCACCAGAACACAGCTGCCTCCAGCCTACACCA
ACAGCTTTACCAGGGGCGTGTACTACCCCGACAAGGTGTTCAGATCCAGCGTGCTGCACTCTACCCAGGACCTGTTCCTGCCTTTCTTC
AGCAACGTGACCTGGTTCCACGTGATTTCCGGCACCAATGGCACCAAGAGATTCGACAACCCCGTGCTGCCCTTCAACGACGGGGTG
TACTTTGCCAGCATCGAGAAGTCCAACATCATCCGCGGCTGGATCTTCGGCACCACACTGGATAGCAAGACCCAGAGCCTGCTGATC
GTGAACAACGCCACCAACGTGGTCATCAAAGTGTGCGAGTTCCAGTTCTGCAACTACCCCTTCCTGGACCACAAGAACAACAAGAGC
TGGACCGAGAGCGAGTTCCGGGTGTACAGCAGCGCCAACAACTGCACCTTCGAGTACGTGTCCCAGCCTTTCCTGATGGACCTGGAA
GGCAAGCAGGGCAACTTCAAGAACCTGCGCGAGTTCGTGTTCAAGAACATCGACGGCTACTTCAAGATCTACAGCAAGCACACCCCT
ATCATCGTGCGGGAACCCGAAGATCTGCCTCAGGGCTTCTCTGCTCTGGAACCCCTGGTGGATCTGCCCATCGGCATCAACATCACCC
GGTTTCAGACACTGCTGGCCCTGCACAGAAGCTACCTGACACCTGGCGATAGCAGCAGCGGATGGACAGCTGGTGCCGCCGCTTACT
ATGTGGGCTACCTGCAGCCTAGAACCTTCCTGCTGAAGTACAACGAGAACGGCACCATCACCGACGCCGTGGATTGTGCTCTGGATC
CTCTGAGCGAGACAAAGTGCACCCTGAAGTCCTTCACCGTGGAAAAGGGCATCTACCAGACCAGCAACTTCCGGGTGCAGCCCACC
GAATCCATCGTGCGGTTCCCCAATATCACCAATCTGTGCCCCTTCGACGAGGTGTTCAATGCCACCAGATTCGCCTCTGTGTACGCCTG
GAACCGGAAGCGGATCAGCAATTGCGTGGCCGACTACTCCGTGCTGTACAATCTGGCCCCCTTCTTCACCTTCAAGTGCTACGGCGTG
TCCCCTACCAAGCTGAACGACCTGTGCTTCACAAACGTGTACGCCGACAGCTTCGTGATCCGGGGAGATGAAGTGCGGCAGATTGCC
CCTGGACAGACAGGCAACATCGCCGACTACAACTACAAGCTGCCCGACGACTTCACCGGCTGCGTGATCGCTTGGAACAGCAACAA
GCTGGACAGCAAAGTCAGCGGCAACTACAATTACCTGTACCGGCTGTTCCGGAAGTCCAATCTGAAGCCCTTCGAGCGGGACATCTC
CACCGAGATCTATCAGGCCGGCAACAAGCCTTGTAACGGCGTGGCCGGATTTAACTGTTATTTTCCCCTTCGGTCCTACAGCTTTCGGC
CCACATACGGCGTCGGCCACCAGCCTTACAGAGTGGTGGTGCTGAGCTTCGAGCTGCTGCATGCTCCTGCCACAGTGTGCGGCCCTAA
GAAAAGCACCAATCTCGTGAAGAACAAATGCGTGAACTTCAACTTCAACGGCCTGAAGGGCACCGGCGTGCTGACAGAGAGCAACA
AGAAGTTCCTGCCATTCCAGCAGTTCGGCCGGGATATCGCCGATACCACAGATGCCGTCAGAGATCCCCAGACACTGGAAATCCTGG
ACATCACCCCTTGCAGCTTCGGCGGAGTGTCTGTGATCACCCCTGGCACCAACACCAGCAATCAGGTGGCAGTGCTGTACCAGGGCG
TGAACTGTACAGAGGTGCCAGTGGCCATCCATGCCGATCAGCTGACCCCTACTTGGAGAGTGTACTCCACCGGCAGCAATGTGTTTCA
GACCAGAGCCGGCTGTCTGATCGGAGCCGAGTACGTGAACAATAGCTACGAGTGCGACATCCCCATCGGCGCTGGCATCTGCGCCTC
TTACCAGACACAGACAAAGAGCCACAGACGGGCCAGATCTGTGGCCAGCCAGAGCATCATTGCCTACACAATGTCTCTGGGCGCCG
AGAACTCTGTGGCCTACTCCAACAACTCTATCGCTATCCCCACCAACTTCACCATCAGCGTGACCACAGAGATCCTGCCTGTGTCCAT
GACCAAGACCAGCGTGGACTGCACCATGTACATCTGCGGCGATTCCACCGAGTGCTCCAACCTGCTGCTGCAGTACGGCAGCTTCTG
CACCCAGCTGAAGAGAGCCCTGACAGGGATCGCCGTGGAACAGGACAAGAACACCCAAGAGGTGTTCGCCCAAGTGAAGCAGATC
TACAAGACCCCTCCTATCAAGTACTTCGGCGGCTTCAATTTCAGCCAGATTCTGCCCGATCCTAGCAAGCCCAGCAAGCGGAGCTTCA
TCGAGGACCTGCTGTTCAACAAAGTGACACTGGCCGACGCCGGCTTCATCAAGCAGTATGGCGATTGCCTGGGCGACATTGCCGCCA
GGGATCTGATTTGCGCCCAGAAGTTTAAGGGACTGACAGTGCTGCCTCCTCTGCTGACCGATGAGATGATCGCCCAGTATACAAGCG
CCCTGCTGGCCGGCACAATCACAAGCGGCTGGACATTTGGAGCTGGCGCCGCTCTGCAGATCCCCTTTGCTATGCAGATGGCCTACAG
ATTCAACGGCATCGGAGTGACCCAGAATGTGCTGTACGAGAACCAGAAGCTGATCGCCAACCAGTTCAACAGCGCCATCGGCAAGA
TCCAGGACAGCCTGAGCAGTACAGCCAGCGCTCTGGGAAAGCTGCAGGACGTGGTCAACCACAATGCCCAGGCACTGAACACCCTG
GTCAAGCAGCTGAGCAGCAAGTTCGGCGCCATCAGCTCTGTGCTGAACGATATCTTCAGCAGACTGGACAAGGTGGAAGCCGAGGT
GCAGATCGACAGACTGATCACCGGAAGGCTGCAGTCCCTGCAGACCTACGTTACCCAGCAGCTGATCAGAGCCGCCGAGATTAGAG
CCTCTGCCAATCTGGCCGCCACAAAGATGAGCGAGTGTGTGCTGGGCCAGAGCAAGAGAGTGGACTTCTGCGGAAAGGGCTACCAC
CTGATGAGCTTCCCTCAGTCTGCACCACACGGCGTGGTGTTTCTGCACGTGACATACGTGCCCGCTCAAGAGAAGAACTTCACAACAA
GCCCCGCCATCTGCCACGACGGCAAAGCCCACTTTCCTAGAGAAGGCGTGTTCGTGTCCAACGGCACCCATTGGTTCGTGACCCAGC
GGAACTTCTACGAGCCCCAGATCATCACCACCGACAACACCTTCGTGTCTGGCAACTGCGACGTCGTGATCGGAATTGTGAACAATA
CCGTGTACGACCCTCTGCAGCCCGAGCTGGACAGCTTCAAAGAGGAACTGGATAAGTACTTTAAGAACCACACTAGCCCCGACGTGG
ACCTGGGCGATATCAGCGGAATCAATGCCAGCGTCGTGAACATCCAGAAAGAGATCGACCGGCTGAACGAGGTGGCCAAGAATCTG
AACGAGAGCCTGATCGACCTGCAAGAACTGGGGAAGTACGAGCAGTATATTAAGTGGCCTTGGTACATCTGGCTGGGCTTTATCGCC
GGACTGATTGCCATCGTGATGGTCACAATCATGCTGTGCTGCATGACCAGCTGCTGTAGCTGCCTGAAGGGCTGTTGTAGCTGTGGCT
CCTGCTGCTGA
